# Multimodal magnetic resonance imaging on brain structure and function changes in subjective cognitive decline: a mini-review

**DOI:** 10.3389/fnagi.2023.1259190

**Published:** 2023-09-18

**Authors:** Jinhuan Yue, Shengwang Han, Ang Li, Zeyi Wei, Danna Cao, Shenglan Gao, Xiaoling Li, Guanhu Yang, Qinhong Zhang

**Affiliations:** ^1^Shenzhen Frontiers in Chinese Medicine Research Co., Ltd., Shenzhen, China; ^2^Department of Acupuncture and Moxibustion, Vitality University, Hayward, CA, United States; ^3^Third Ward of Rehabilitation Department, Second Affiliated Hospital of Heilongjiang University of Chinese Medicine, Harbin, China; ^4^Servier (Beijing) Pharmaceutical Research & Development CO., Ltd., Beijing, China; ^5^Graduate School of Heilongjiang University of Chinese Medicine, Harbin, China; ^6^Division of CT and MRI, First Affiliated Hospital of Heilongjiang University of Chinese Medicine, Harbin, China; ^7^Department of Specialty Medicine, Ohio University, Athens, OH, United States; ^8^Heilongjiang University of Chinese Medicine, Harbin, China

**Keywords:** subjective cognitive decline, multimodal, magnetic resonance imaging, brain structure, brain function

## Abstract

Subjective cognitive decline (SCD) is the initial stage of Alzheimer’s disease (AD). Early identification of SCD and its risk factors is of great importance for targeted interventions and for delaying the onset of AD. We reviewed the relevant literature on structural magnetic resonance imaging (sMRI), diffusion tensor imaging (DTI), functional magnetic resonance imaging (fMRI), and other techniques regarding SCD research in recent years. This study applied sMRI and fMRI techniques to explore abnormal brain structures and functions, which may help provide a basis for SCD diagnosis.

## Introduction

Subjective cognitive decline (SCD) refers to self-reported experiences of memory loss and is considered as the initial stage of Alzheimer’s disease (AD; Taylor et al., 2018; Gaugler et al., 2020). Mild cognitive impairment (MCI) is a clinical stage between normal aging and AD and serves as a precursor to AD after SCD, with an annual conversion rate to MCI of 10–15% (Arvanitakis et al., 2019). The National Institute on Aging-Alzheimer’s Association group has standardized the evaluation and biomarker analysis of AD. They suggest that during the SCD stage, there is no cognitive impairment, while in the MCI stage, there is evidence of memory or other cognitive domain impairment. Biomarker analyses have confirmed that AD can cause dementia (Albert et al., 2011; Jack et al., 2011; McKhann et al., 2011; Sperling et al., 2011). During the MCI stage, individuals may experience subtle cognitive changes that do not significantly affect their daily activity. Previous studies have suggested that prodromal AD symptoms can persist for 15–20 years (Villain et al., 2012; Jack et al., 2013; Villemagne et al., 2013; Villeneuve et al., 2015), and SCD patients are highly likely to progress to MCI or further AD (Jessen et al., 2014). The SCD is considered an early factor related to AD biomarker abnormalities, future decline in cognitive abilities, and an increased risk of AD (Jessen et al., 2014; Jack et al., 2018; Slot et al., 2019). Therefore, studying SCD is crucial for understanding the early pathological mechanisms of AD and identifying the associated biomarkers.

Magnetic resonance imaging (MRI) has revolutionized the field of neuroscience by providing a non-invasive method for evaluating the structure, function, and neurochemistry of the human brain (Achard and Bullmore, 2007). By correlating brain measurements with behavioral outcomes or clinical symptoms, researchers can infer the potential mechanisms underlying clinical differences (Brown et al., 2016). Structural MRI (sMRI), diffusion tensor imaging (DTI), and functional MRI (fMRI) can be used to detect changes in the brain structure and function associated with SCD.

## Brain structure changes in SCD

**Figure 1:**
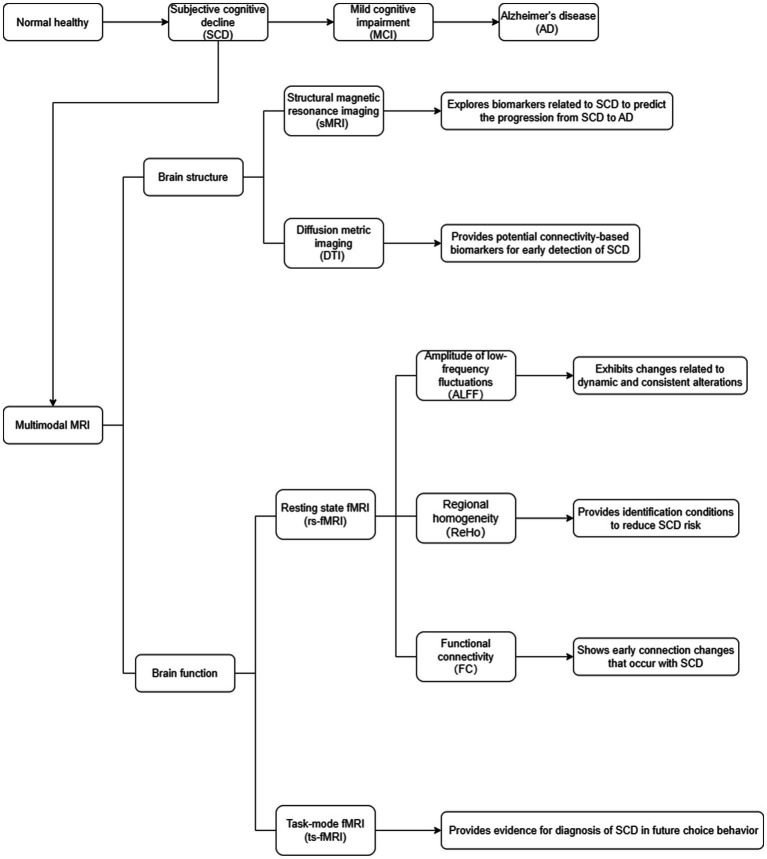
Multimodal MRI of brain structure and function in SCD.

The sMRI can clearly display and obtain high-resolution images of human brain structures. Many studies have used sMRI as the basis for exploring biomarkers related to SCD (Figure 1). By comparing the differences between the brain and actual age, it can better predict the progression from SCD to AD (Wang M. et al., 2022; Wang et al., 2023).

The DTI is commonly used in MRI to describe the complex white matter fiber bundle structure in the human brain (Wu et al., 2018). A domain-prior-induced structural MRI adaptation (DSMA) method was applied to predict the progression of SCD by alleviating the distribution discrepancy between SCD and AD groups (Yu et al., 2022). This method consists of two parallel feature encoders, which are used to locate the attention blocks in the brain region related to potential diseases in both the labeled source domain and the unlabeled target domain, and a feature adaptation module based on the maximum mean discrepancy for cross-domain feature alignment. Experimental results on the public AD Neuroimaging Initiative and SCD datasets showed that the DSMA method outperformed existing techniques. The DTI and graph theory analyses were utilized to investigate whether SCD patients exhibit disruption in white matter connectivity and topological changes in brain structural networks (Shu et al., 2018). The graph theory analysis results showed that SCD patients had lower overall efficiency and local efficiency than the healthy control group. Lower regional efficiency was mainly distributed in the bilateral frontal lobes and left thalamus. This study confirmed that the topological efficiency of brain structural networks is disrupted in patients with SCD and provides potential connectivity-based biomarkers for the early detection of SCD.

A previous study extracted grey matter volume from 10 easily identifiable regions of interest that are associated with higher-order cognitive functions and dysfunction in AD-related functions (Fu et al., 2021). The volume values were then used to predict regional connectivity across the entire brain using voxel-based statistical models and multivariate linear regression models. In comparison to cognitively normal individuals, individuals with SCD showed reduced structural covariance and weakened connectivity (Fu et al., 2021). Structural covariance networks (SCNs) generated from the Default Mode Network, Salience Network, hippocampal subregions, and cholinergic basal forebrain displayed increased covariance in individuals with early-stage AD (amnestic MCI, aMCI), while decreased covariance was observed in individuals in the dementia stage of AD. Furthermore, SCNs seeded from the Executive Control Network showed a linear increase in structural covariance magnitude in both early-stage and dementia stages. The findings of this study suggest that the changes in structural covariance patterns in the normal individual-SCD-aMCI-AD progression are divergent and dynamic, providing support for the hypothesis of structural disconnection in individuals with SCD (Fu et al., 2021).

## Brain function changes in SCD

The fMRI can be divided into resting-state functional MRI (rs-fMRI) and task-state MRI (ts-fMRI; Sun et al., 2021). The rs-fMRI involves a state of relaxation in which subjects close their eyes and avoid any structural thought activity as much as possible using blood oxygen level-dependent (BOLD) signals to measure changes in brain function (Ten Kate et al., 2018). The rs-fMRI technique mainly includes amplitude of low-frequency fluctuations (ALFF), regional homogeneity (ReHo), and functional connectivity (FC; Figure 1; Li et al., 2021; Hao et al., 2022). The ts-fMRI involves giving subjects certain task stimuli while simultaneously performing BOLD-fMRI signal acquisition and analyzing the changes in the functional state of each brain area during the task state (Figure 1; Kang et al., 2021).

A previous study applied rs-fMRI to investigate the dynamic and consistent changes in brain activity indices in individuals with SCD (Yang et al., 2021). The results showed that individuals with SCD had lower values of ALFF in bilateral hippocampus (HP)/parahippocampal gyrus (PHG)/fusiform gyrus (FG), and bilateral cerebellum, as well as lower values of ALFF in the bilateral precuneus and posterior cingulate gyrus. In contrast, individuals with MCI had higher ALFF. Consistency in the bilateral HP/PHG/FG, temporal lobes, and left inferior frontal cortex was higher in individuals with SCD than in normal controls, whereas consistency was lower in individuals with MCI. Brain activity in individuals with SCD exhibited changes related to dynamic and consistent alterations, suggesting the presence of compensatory mechanisms in these individuals.

A published study evaluated the relationship between social relationships (close friends), subthreshold geriatric depression (SGD), and SCD from a brain functional perspective (Zhang et al., 2022). The ReHo of fMRI data was calculated for each participant. After correction for multiple comparisons, the right posterior cingulate gyrus (SOG.R) and right fusiform gyrus (FFG.R) were found to be positively correlated with SGD in individuals with SCD. These findings indicate that social relationships modulate the functionality of specific brain regions, and that SGD may be an early symptom of SCD. Furthermore, FFG.R mediates the relationship between social relationships and SGD, whereas abnormalities in SOG.R may be a key factor in depression-induced SCD. Moreover, having close friends can reduce the risk of developing SGD.

One study used a sliding window, Pearson correlation, and cluster analysis in rs-fMRI to investigate the transformation of FC strength information between different brain regions in individuals with SCD, specifically in low, intermediate, and high states (Wang J. et al., 2022). The results showed that the dynamic functional state changes in SCD and AD patients were primarily observed in the high-intensity FC state. Therefore, a highly connected state measured by dynamic FC may represent an early change in AD. Based on the findings by Herdick et al. (2020), they used FC analysis to compare different groups. They found that in individuals with SCD, there were no significant differences in FC in the anterior cholinergic basal forebrain compared to healthy control group. However, in the MCI group, there was a decrease in connectivity in 18 voxels in the right medial frontal gyrus. On the other hand, individuals with AD dementia showed a greater decrease in connectivity in 74 voxels in the bilateral pregenual anterior cingulate cortex and right medial frontal gyrus. When it comes to the posterior-lateral basal forebrain, there were no significant differences in FC between the SCD and healthy control groups. The MCI group showed two small clusters of decreased connectivity in the right mid superior frontal gyrus (13 voxels) and right superior temporal gyrus (23 voxels). AD dementia subjects displayed two small clusters of decreased connectivity in the left pregenual anterior cingulate cortex (23 voxels) and right parahippocampal gyrus (18 voxels). Therefore, compared to the healthy controls, both MCI and AD dementia showed significant differences in the volume and mean diffusivity of the cholinergic basal forebrain. However, there were no significant differences in these measures between the SCD and healthy control groups.

A ts-fMRI study on intertemporal decision-making in participants with SCD in the absence of future imagined scenarios (Hu et al., 2017). The results show that participants with SCD had fewer future-oriented choices. Future imagination increased future-oriented choices and was only associated with increased brain activation in the medial prefrontal cortex, right insular cortex, and anterior cingulate cortex in the control group but not in individuals with SCD. Furthermore, more future-oriented choices were associated only with hippocampal activation during choice processing in the control group. These findings suggest that subtle disruptions in the neural networks in SCD may underlie their short sightedness in future decision-making and the lack of regulation of choice behavior through prospective imagination scenarios.

## Limitations

There are several factors that contribute to the limitations and challenges associated with the use of MRI in research and medical settings. The first major hurdle is the high costs associated with MRI scans. The expense of the equipment itself, maintenance costs, and the need for highly trained personnel all contribute to the high costs of MRI scans. This can limit the accessibility of MRI technology, particularly in resource-limited areas or low-income communities. Another challenge is the difficulty in combining MRI with cognitive testing. MRI scans provide detailed structural and functional information about the brain, but performing cognitive tasks inside the scanner can be challenging due to the confined space and the need to limit head movements. This can make it difficult to accurately assess cognitive functions in real-time during MRI scans, limiting the ability to correlate cognitive performance with brain activity. Furthermore, MRI suffers from low temporal resolution, which refers to the ability to accurately capture the timing of neuronal communication. MRI scans typically take several seconds to capture an image, making it difficult to detect rapid changes or transient brain events that occur within milliseconds. This temporal mismatch between MRI scans and the timing of neuronal communication poses a challenge for studying dynamic brain processes. Additionally, there is a lack of agreed-upon gold-standard and quality control protocols for MRI. The field of MRI research and clinical practice is still evolving, and there is a need for standardized protocols that ensure consistency and reliability across different studies and healthcare centers. This lack of standardized protocols can lead to variations in data acquisition, analysis, and interpretation, making it challenging to compare results across studies or establish universally accepted guidelines for MRI usage. Overall, while MRI is a powerful tool for investigating the brain, it faces limitations such as high costs, difficulties in combining with cognitive testing, low temporal resolution, and the lack of agreed-upon gold-standard protocols. Addressing these challenges and developing innovative solutions will be crucial for advancing the field and making MRI more accessible and effective in both research and clinical settings.

## Summary

AD research has gradually progressed from the application of sMRI to fMRI, with the aim of the early diagnosis of SCD and prevention of AD occurrence, providing assistance in clinical diagnosis and treatment. Based on MRI, abnormalities in brain structure and function can be detected in SCD, specifically manifested as damage to the topological efficiency of brain structural connections, changes in ALFF, ReHo values, FC intensity, and abnormal brain region activation. These findings contribute to the exploration of the central mechanisms related to SCD and provide imaging evidence for diagnosis and treatment.

Future studies should focus more on the MRI in SCD, aiming to establish comprehensive diagnostic models that can accurately assess the disease progression and its influencing factors in the SCD population. These studies should also involve conducting clinical trials for interventions across different fields, providing further insights into the pathological and physiological mechanisms underlying SCD. The research should involve longitudinal and in-depth investigation, with the ultimate goal of exploring the shifting paradigm of SCD.

## Author contributions

JY: Conceptualization, Validation, Visualization, Writing – original draft, Writing – review & editing. SH: Funding acquisition, Validation, Visualization, Writing – original draft, Writing – review & editing. AL: Methodology, Validation, Visualization, Writing – original draft, Writing – review & editing. ZW: Conceptualization, Data curation, Resources, Validation, Visualization, Writing – original draft, Writing – review & editing. DC: Funding acquisition, Validation, Visualization, Writing – original draft, Writing – review & editing. SG: Resources, Validation, Visualization, Writing – original draft, Writing – review & editing. QZ: Conceptualization, Data curation, Investigation, Project administration, Supervision, Validation, Visualization, Writing – original draft, Writing – review & editing. XL: Resources, Supervision, Validation, Visualization, Writing – original draft, Writing – review & editing, Conceptualization, Data curation, Funding acquisition, Investigation, Project administration. GY: Investigation, Project administration, Supervision, Validation, Visualization, Writing – original draft, Writing – review & editing.

## Funding

The author(s) declare financial support was received for the research, authorship, and/or publication of this article. This study was partly funded by the National Foundation of Natural Science of China (82074537, 81373714), Joint Guidance Project of Natural Science Foundation of Heilongjiang Province (LH2020H103, LH2021H101), and Research Projects of the Chinese Medicine Administration of Heilongjiang (ZHY2022-194). The funder had no roles in this study.

## Conflict of interest

JY and QZ are employed by Shenzhen Frontiers in Chinese Medicine Research Co., Ltd. and AL is employed by Servier (Beijing) Pharmaceutical Research & Development CO. Ltd.

The remaining authors declare that the research was conducted in the absence of any commercial or financial relationships that could be construed as a potential conflict of interest.

## Publisher’s note

All claims expressed in this article are solely those of the authors and do not necessarily represent those of their affiliated organizations, or those of the publisher, the editors and the reviewers. Any product that may be evaluated in this article, or claim that may be made by its manufacturer, is not guaranteed or endorsed by the publisher.

## Figures and Tables

**Table 1:** 

SCD	Subjective cognitive decline
AD	Alzheimer’s disease
sMRI	Structural magnetic resonance imaging
DTI	Diffusion tensor imaging
fMRI	Functional magnetic resonance imaging
MCI	Mild cognitive impairment
MRI	Magnetic resonance imaging
DSMA	Domain-prior-induced structural MRI adaptation
SCNs	Structural covariance networks
aMCI	Amnestic MCI
rs-fMRI	Resting-state functional magnetic resonance imaging
ts-fMRI	Task-state magnetic resonance imaging
BOLD	Blood oxygen level dependent
ALFF	Amplitude of low-frequency fluctuations
ReHo	Regional homogeneity
FC	Functional connectivity
HP	Hippocampi
PHG	Bilateral parahippocampal gyri
FG	Fusiform gyri
SGD	Subthreshold geriatric depression
SOG.R	Right posterior cingulate gyrus
FFG.R	Right fusiform gyrus
